# May God Guide Our Guns: Visualizing Supernatural Aid Heightens Team Confidence in a Paintball Battle Simulation

**DOI:** 10.1007/s12110-018-9320-8

**Published:** 2018-06-18

**Authors:** Jeremy Pollack, Colin Holbrook, Daniel M. T. Fessler, Adam Maxwell Sparks, James G. Zerbe

**Affiliations:** 1 California State University, Dominguez Hills, Carson, CA USA; 2 Department of Cognitive and Information Sciences, University of California, Merced, 5200 N. Lake Rd., Merced, CA 95343 USA; 3 University of California, Los Angeles, Los Angeles, CA USA; 4 Arizona State University, Tempe, AZ USA

**Keywords:** Religion, Violence, Team sports, Threat, Aggression, Coalitional psychology

## Abstract

The perceived support of supernatural agents has been historically, ethnographically, and theoretically linked with confidence in engaging in violent intergroup conflict. However, scant experimental investigations of such links have been reported to date, and the extant evidence derives largely from indirect laboratory methods of limited ecological validity. Here, we experimentally tested the hypothesis that perceived supernatural aid would heighten inclinations toward coalitional aggression using a realistic simulated coalitional combat paradigm: competitive team paintball. In a between-subjects design, US paintball players recruited for the study were experimentally primed with thoughts of supernatural support using a guided visualization exercise analogous to prayer, or with a control visualization of a nature scene. The participants then competed in a team paintball battle game modeled after “Capture the Flag.” Immediately before and after the battle, participants completed surveys assessing confidence in their coalitional and personal battle performance. Participants assessed their coalition’s prospects of victory and performance more positively after visualizing supernatural aid. Participants primed with supernatural support also reported inflated assessments of their own performance. Importantly, however, covarying increases in assessments of their overall coalition’s performance accounted for the latter effect. This study provided support for the hypothesis that perceived supernatural support can heighten both prospective confidence in coalitional victory and retrospective confidence in the combat performance of one’s team, while highlighting the role of competitive play in evoking the coalitional psychology of intergroup conflict. These results accord with and extend convergent prior findings derived from laboratory paradigms far removed from the experience of combat. Accordingly, the field study approach utilized here shows promise as a method for investigating coalitional battle dynamics in a realistic, experientially immersive manner.

**Electronic supplementary material:**

The online version of this article (10.1007/s12110-018-9320-8) contains supplementary material, which is available to authorized users.

When you go out to war against your enemies, and see horses and chariots and an army larger than your own, you shall not be afraid of them, for the Lord your God is with you (Deuteronomy 20:1, ESV)

Religious conviction has been broadly implicated as a driver of bold, aggressive action in the face of violent opposition. Although some aggressive acts associated with religiosity may be motivated by factors such as moral convictions or societal norms orthogonal to supernatural cognition per se, convergent lines of evidence indicate that perceiving supernatural agents as sources of aid can inspire aggressive responses to conflict by bolstering confidence that one’s group will triumph, much as one would expect of a group with access to powerful natural allies. For example, participants experimentally cued with reminders of either actual or supernatural allies envision a threatening male adversary as being less physically formidable than do controls (Holbrook et al. 2016a), and subliminal primes of religious concepts increase levels of costly punishment in economic games in a manner reminiscent of retributive aggression (McKay et al. 2011). Similarly suggestive that belief in supernatural allies attenuates fear of violent conflict, studies of trait religiosity reveal that faith in supportive supernatural agents negatively correlates both with the fear of death (Jong et al. 2013) and with reactivity to reminders of death (Jonas and Fischer 2006). Conversely, heightened belief in God has been associated with reminders of mortality, consistent with a facultative shift enabling individuals to maintain equanimity despite the prospect of death (Holbrook et al. 2016b; Jong et al. 2013). Further, and consistent with the premise that reduced fear of being harmed or killed can motivate willingness to risk violent confrontation under circumstances of coalitional conflict, trait religiosity tracks propensities for aggression on behalf of in-groups (e.g., Atran and Ginges 2012; Kruglanski et al. 2009; Sosis et al. 2012).

The tendency for belief in supernatural allies to spur aggressive confrontation with opposing groups may appear maladaptive and hence likely to be selected against. However, cultural group selection may exploit the evolved capacity to represent unseen agents as though they were real in a manner that advances the growth of both religious traditions and the societies in which they are embedded. The capacity to represent supernatural agents appears to be a by-product of mental adaptations for social functions such as Theory of Mind (Boyer and Bergstrom 2008; Schjødt et al. 2009). In societies wherein supernatural agents are conceptualized as potentially (parochially) benevolent moral beings, such by-product representations of the mental states, intentions, and so on, of supernatural agents may interdigitate with adaptations related to interpersonal affiliation to enable the representation of invisible agents with whom one may experience a supportive relationship (Holbrook et al. 2015). At the individual level, doctrines involving benevolent supernatural entities may bolster sanguinity in the face of dire challenges; at the group level, communities united by shared religious beliefs, practices, and institutions may be better able to cooperate (Atran and Ginges 2012). In this manner, cultural group selection may favor societies that maintain beliefs in benevolent supernatural agents because such societies appear to benefit from greater cooperation, thereby enhancing the capacity to engage in coordinated action—including warfare (Richerson et al. 2016; Roes and Raymond 2003).

In addition to the many plausible advantages of enhanced cooperation, another pathway by which cultural beliefs in benevolent supernatural agents may benefit groups is by increasing individuals’ confidence in victory, and hence their willingness to engage in intergroup warfare. When aggregated over numerous conflicts—and notwithstanding instances in which overconfidence leads to disastrous defeats—overconfidence in the prospects of winning can engender success in intergroup conflict by intimidating opponents or strengthening resolve to fight (Johnson 2015; Johnson and Fowler 2011; Wrangham 1999). Over time, such a pattern of victories may be expected to enlarge and better resource groups (Roes and Raymond 2003; Turchin 2006). At the individual level, such overconfidence in the face of violence could also yield beneficial outcomes under some circumstances, but it would clearly entail heightened risk of injury or death. However, individual-level selection against the capacity to represent supernatural agents owing to overconfidence-related injury or death would only apply to societies that promulgate belief in supernatural support, and would therefore be offset by the individual-level benefits of enhanced group cooperation enjoyed within such groups. Further, selection against belief in supernatural agents would appear highly constrained to the extent that this representational capacity derives from fundamental, adaptive social cognitive capacities (e.g., Theory of Mind). In sum, despite the inherent risks, individuals should be expected to display confidence in engaging in violent conflict when representing themselves as being in league with powerful supernatural allies, in a pattern likely owing to by-products of adaptations for other social functions, and likely leveraged by forces of cultural group selection (Whitehouse 2004).

Ethnographic research indicates that small-scale societies predominantly lack beliefs in supernatural agents invested in the welfare or moral conduct of human beings (Atran and Ginges 2012). By contrast, in their analysis of data from the *Ethnographic Atlas* (composed of 1267 societies; Gray 1999) and the Standard Cross-Cultural Sample (composed of 186 societies; Murdock and White 1969), Roes and Raymond (2003) found that beliefs regarding gods that monitor moral conduct and actively intervene in human affairs positively correlate with the relative size of societies, which in turn predicts the tendency to engage in intergroup warfare. For example, the rapid rise of early Islam has been attributed, in part, to the interplay between belief in supernatural allies, in-group cooperation, and propensities to engage in and succeed in warfare (Ibrahim 1990).

Here, building on these suggestive patterns, we seek to test whether, in a cultural context in which predominant portrayals of supernatural agents represent them as both benevolent and omnipotent, perceptions of supernatural aid promote battle confidence. Although the ethnographic data broadly accord with the premise that members of groups that maintain belief in supernatural entities who may intervene on their behalf will be relatively confident in the face of battle, these data are historically contingent and involve complex, difficult to disentangle components. For example, does belief in supportive supernatural agents who morally police behavior increase willingness to engage in warfare via cooperation-enhancing pathways (e.g., the deterrence of free-riding) orthogonal to enhanced confidence that supernatural benefactors will bring victory? Surprisingly scant experimental work has examined whether perceptions of supernatural support increase battle confidence, and the extant studies predominantly rely on indirect methods, hypothetical judgments, and/or correlational results pertaining to trait religiosity (correlations that may be due to confounding personality or sociodemographic variables). How, then, to test the hypothesized link between perceived supernatural support and battle confidence in an ecologically valid manner more true to the actual experience of violent coalitional conflict? One strategy may be to capitalize on the evocative power of competitive play.

Play has been understood as the product of adaptations for the preparatory rehearsal of specific survival and reproductive abilities (Smith 1982), notably including combat-relevant behaviors such as fighting, hiding, or fleeing from hostile conspecifics or predators (Boulton and Smith 1992; Scalise Sugiyama et al. 2016; Symons 1978). Given the substantial influence of coalitional violence on fitness in the ancestral past (Bowles 2009; Komar 2008; Manson and Wrangham 1991), and given the complex cognitive and physical demands inherent to coordinating within one’s own coalition while anticipating and effectively countering the strategies of opposing coalitions, engaging in play simulations of competitive group conflict appears to provide a relatively safe, adaptive means of refining skills relevant to coalitional combat that would continue to reap benefits into adulthood. Consonant with this thesis, in the ethnographic record, combative sports—especially sham combat sports that mimic warfare—are more common in societies that engage in warfare (Chick et al. 1997; Sipes 1973), whereas team combative sports in particular are less likely to occur in societies where warfare is absent or rare (Chick et al. 1997). The adaptive advantages of preparatory group play-fighting in the ancestral past may partially explain both the continuing appeal of such activities in contemporary contexts (e.g., team sports, warfare video games), as well as the pattern wherein this particular mode of play often remains compelling into the adult years, long after other forms of play fall by the wayside. However, in addition to a preparation/practice construal, a number of distinct perspectives on play-fighting highlight other plausible benefits, such as facilitating social bonding or establishing social hierarchies (Bekoff and Byers 1981). In our view, these interpretations are mutually compatible inasmuch as skill refinement, group bonding, and effective operation within hierarchies are all integral to effective coalitional combat performance. Accordingly, the proximate experiences of fun, arousal, and engagement associated with competitive intergroup play may relate to such non-preparatory functions, or even to by-product activation of mechanisms evolved for engaging in actual coalitional conflict. For the purposes of the present research, the salient claim is not that group play-fighting serves a particular set of ultimate functions, but that it evokes mechanisms intrinsic to the psychology of coalitional warfare. To exploit this affordance of play, we conducted our experimental test of the effect of cues of supernatural support in the context of competitive team paintball.

Paintball has been frequently employed to realistically simulate combat for purposes of police and military training because of its faithful recreation of the experience of gun combat in urban settings, including a quick, chaotic pace, and the genuine stakes posed by the aversive pain of being hit (Spaulding 2005). We staged team-based paintball battles, experimentally primed these teams with either cues of supernatural support or a control topic, and solicited participants’ ratings of confidence in their coalition’s prospects for victory, in the overall performance of their coalitions, and in their personal performance. These measures were administered both immediately before and immediately after battle. Consonant with the hypothesis that perceived supernatural support enhances battle confidence, we predicted that players in the supernatural support condition would exhibit greater *pre-battl*e confidence, specifically with regard to:

1. Anticipation of victory
2. Anticipation of their personal battle performance
3. Anticipation of their coalition’s battle performance

Likewise, we predicted that players in the supernatural support condition would exhibit greater confidence *post-battle* with regard to:

4. Subjective feeling of victory (as designed, objective victory was essentially impossible)
5. Anticipation of future victory
6. Appraisal of their personal battle performance
7. Appraisal of their coalition’s battle performance

In addition to these predictions, we also explored possible effects of the supernatural-support manipulation on battle-related emotion. Finally, although we did not measure actual battlefield behavior in this field study, we did include an exploratory self-report measure of risky behavior (i.e., crossing open ground rather than taking cover).

## Methods

The research team partnered with a retail paintball supplies company to conduct a field study coinciding with a regularly scheduled monthly play event. In the context of an active commercial paintball event, the research team recruited individuals for the study in exchange for free play for the day (a $25 value) as well as five tickets for a raffle of paintball equipment prizes to be held at the end of the day. Fifty-nine players were recruited, and then filtered for response completeness, because investing the time to answer the questions in the pre-battle survey and returning later to fill out the post-battle survey indicates attention and sincerity. This completeness criterion yielded a final sample of 46 players (100% male).^1^ We did not drop players based on completion of a demographics packet administered last in the study sequence since our predictions do not involve age or ethnicity, and since many players likely failed to return to the survey-collection site for the demographic questions owing to the somewhat chaotic environment of a large paintball event (more on this below). Accordingly, demographic data were collected for only 26 players, for whom the following obtains: age range 18 to 55 (*M* = 26.8; *SD* = 6.93); 61.5% Latino, 19.2% White, 7.7% Asian; 3.8% Black, 7.7% Other).

**Fig. 1:**
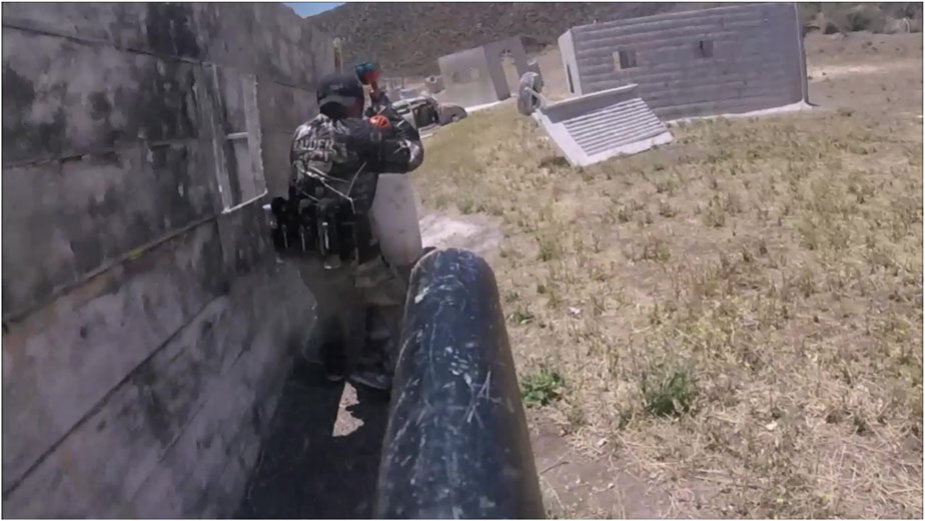
Photograph conveying the participant’s point of view during simulated coalitional combat at the paintball event on the date of data collection. Image shot with a head-mounted camera shortly before data collection commenced. Video available at https://osf.io/e9d68/?view_only=85cba56aba3e4a7cbe9d651d5f654d9e

The study was framed as ostensibly exploring the impact of mental visualization on athletic performance, with no mention of religiosity. Players were split into four squads of ~15 members, each of which competed in one of two five-minute games of “Capture the Flag.” Each squad was led to opposite ends of a large outdoor paintball arena that is staged so as to resemble an urban warfare context. Squads were then informed of the game rules: to win, a squad member must capture the opposing squad’s flag and return with it to their side of the field. These game rules were selected because victory for either side within five minutes would be extremely unlikely. Thus, in the absence of objective victory (i.e., in the near-certain event of a draw between the two squads), the effect of the supernatural support manipulation on players’ subjective perceptions of their squad’s performance might be more cleanly assessed (for a still image and a link to a video of paintball play during the event, see Fig. 1).

In a between-subjects design, players were randomly assigned to a supernatural-support versus control condition. Participants were randomly assigned to their teams and were assigned blue or gold bracelets with ID numbers to ensure accurate encoding of their experimental condition and accurate pairing of their pre- and post-battle survey packets. The colored bracelets were also intended to reinforce a sense of team identity, which was plausibly further reinforced by the fact that teammates were wearing closely similar paintball gear and facemasks, that they were addressed collectively by research assistants as either “Blue Squad” or “Gold Squad,” that each team was escorted to the battle area as a group, and that, as described below, participants engaged in the experimentally manipulated visualization activity together and in the same physical posture. The fact that they were engaging in a competitive team activity further reinforced the salience of their membership within teams.

Perceived supernatural support was manipulated via an audio recording described as “a visualization exercise that may help in this kind of challenge.” The supernatural visualization primed players (*N* = 22) to envision support from unseen powers (i.e., “God, or spirit, or the universe”) protecting and guiding them during the battle; the control visualization (*N* = 24) involved vividly imagining a tree (see Electronic Supplementary Materials [ESM] for the full text and to access the audio stimuli). The nature of the unseen supernatural support was left intentionally vague in order to engage persons of differing faiths as well as avowed non-believers. Focus-group tests of these stimuli prior to the study confirmed that US participants would find both visualizations engaging, and that the supernatural visualization would convey a sense of the presence of supportive supernatural agents or forces. The visualizations were presented in counterbalanced order and played aloud using portable speakers. Given that group prayer can effectively prime supernatural agency (Bremner et al. 2011), we asked players in each group to listen to the visualizations while “taking a knee” with their eyes closed just prior to the imminent battle, in a circumstance analogous to group prayer.

Players received the pre-battle survey immediately after the visualization exercise. A series of face-valid, 8-point Likert ratings probed confidence regarding the upcoming battle (1 = Not at all; 8 = Extremely). *Anticipated victory* was measured according to responses to “How confident are you that your squad will WIN (i.e., capture the flag)?” We also probed anticipation that the squad would avoid defeat: “How confident are you that your squad will NOT LOSE (i.e., win or draw)?” *Personal confidence* was measured via responses to “How well do you feel YOU will perform in the battle compared with members of the opposing squad?” *Coalitional confidence* was measured using responses to “How well do you feel your SQUAD will perform compared with the opposing squad?” Finally, to assess the extent to which the supernatural prime might influence personal confidence in a manner driven by confidence in the coalition of which the participant was a member, we asked, “How well do you feel that YOU will perform in the game compared with the other members of your squad?” In exploratory questions intended to probe potential effects of the manipulation on battle-related affect, we also probed feelings of excitement or nervousness, using the same scale anchors (see ESM).^2^

Following the battle, the players were led back to their squad’s side of the arena to complete a post-battle survey. *Perceived victory* was measured according to responses to the question “In the battle, did your squad win, lose, or draw?” (Since no participants selected “lose,” these responses were coded as 1 = Draw; 2 = Win.) Next, participants answered items closely paralleling the pre-battle confidence measures, rephrased as past-tense appraisals of the battle that had just transpired (1 = Not at all; 8 = Extremely). *Personal performance* was appraised via responses to “How well do you feel YOU performed in the battle compared with members of the opposing squad?” *Coalitional performance* was measured according to responses to “How well do you feel your SQUAD performed in the battle compared with the opposing squad?” As previously, to assess the extent to which the supernatural prime might influence appraisals of personal performance in a manner independent of perceived coalitional performance during the battle, we asked, “How well do you feel YOU performed in the battle compared with the other members of your squad?” *Rematch confidence* was measured according to responses to “If there were a rematch, how confident are you that your squad would win?” Also as before, exploratory questions probed potential effects of the manipulation on battle-related anxiety or excitement (see ESM).

To ensure that any apparent effects of the supernatural manipulation on post-battle performance appraisals were not artifacts of randomly occurring differences in actual performance between the two conditions (e.g., players who were not shot might perceive their performance as having been superior for reasons orthogonal to envisioned supernatural support), we also asked players to report how many enemies they had hit and whether or not they had been shot during the battle. Finally, in a self-report assessment of potential effects of perceived supernatural support on aggressive risk-taking during the battle simulation, players answered: “During the game, which strategy did you use the most? (0 = Take cover and target opponents; 1 = Cross open ground to capture the enemy flag).

Once the series of battles had concluded, those players who remained in the vicinity were given a demographic packet. Not all participants remained because the circumstances of data collection in the large, open area were relatively chaotic in comparison to laboratory research, reflective of the significant background noise and distraction of other ongoing paintball contests.^3^

## Results

The full dataset and analysis syntax for this study can be accessed at https://osf.io/e9d68/?view_only=85cba56aba3e4a7cbe9d651d5f654d9e. The full study materials are available in the ESM.

### Self-Reported State Affect

Participants in both conditions reported low to moderate anxiety and moderate to high levels of excitement both before and after battle (see ESM Table S1).

#### Null Effects of Supernatural Visualization on State Affect

Analyses of variance revealed no significant effects of condition on the exploratory self-report measures of pre- or post-battle anxiety or excitement, with *p* values ranging from .34 to .97, indicating that the observed effects of the supernatural visualization were independent of effects on consciously reportable affect. Accordingly, state affect is not considered in subsequent analyses.

### Supernatural Visualization and Measures of Pre-battle Confidence

We first tested Hypothesis 1, that the supernatural visualization would heighten anticipation of victory. Consistent with predictions, an analysis of variance revealed that players in the supernatural condition showed greater confidence that their team would win relative to the players in the control condition (Table 1). Unexpectedly, however, there was no complementary effect of the supernatural visualization on expectations that the players’ squad would *avoid* a draw or a defeat, *p* = .53. Despite their logical equivalence (i.e., winning entails avoiding a draw or a defeat), responses to these two measures were only mildly and nonsignificantly correlated, *r*_44_ = .26, *p* = .08, suggesting that participants processed the two questions rather differently.

**Table 1:** Effects of experimental condition on measures of pre-battle and post-battle confidence (*N* = 46)

Hypotheses	Control *Mean* (*SD*)	Supernatural *Mean* (*SD*)	*F*	*p*	η^2^_*p*_	95% CI
Pre-battle						
H1. Anticipated victory	6.17 (1.90)	7.41 (1.10)	7.18	.010	.14	.31, 2.18
H2. Personal performance	6.54 (1.67)	6.73 (1.20)	.18	.670	.00	−.69, 1.06
H3. Coalitional performance	6.38 (1.84)	6.73 (1.61)	.48	.494	.01	−.68, 1.38
Post-battle						
H4. Perceived victory	1.04 (.20)	1.50 (.51)	16.43	<.001	.27	.23, .69
H5. Rematch confidence	5.58 (1.91)	7.09 (1.54)	8.59	.005	.16	.47, 2.54
H6. Personal performance	5.17 (1.93)	6.77 (1.45)	10.08	.003	.19	.59, .63
H7. Coalitional performance	4.88 (2.01)	6.86 (1.42)	14.77	.001	.25	.95, 3.03

“Personal performance” refers to ratings of the self relative to opposing players

We next tested Hypothesis 2, that the supernatural visualization would heighten participants’ expectations of their personal performance during the battle. Departing from expectations, we observed no effect of the supernatural visualization on players’ pre-battle confidence in their personal battle performance relative to the performance of opposing players (Table 1).

Next, we tested Hypothesis 3, that the supernatural visualization would heighten participants’ expectations of their coalition’s battle performance. Again departing from expectations, we observed no effect of the visualization condition on anticipated coalitional battle performance (Table 1). Finally, there was also no effect of the supernatural visualization in our exploratory assessment of ratings of players’ anticipated personal performance relative to their fellow squad members (Supernatural: *M* = 6.09, *SD* = 1.69; Control: *M* = 6.25, *SD* = 1.85), *p* = .763. Mean levels of self-reported confidence were high with respect to both anticipated coalitional and personal performance in both conditions (Table 1).

### Supernatural Visualization and Measures of Post-battle Confidence

As intended, no team captured their opponent’s flag, and hence, viewed objectively, there were no victors. Despite this, as predicted in Hypothesis 4, players exposed to the supernatural visualization perceived their squad as having won the battle (Table 1). Also as predicted in Hypothesis 5, participants in the supernatural visualization condition rated their squad as having a greater chance of winning in a rematch (Table 1). We next tested our predictions with regard to appraisals of performance during the battle. Consistent with Hypothesis 6, participants exposed to the supernatural visualization appraised themselves as having personally performed more skillfully than the opposing players (Table 1). Finally, consistent with Hypothesis 7, participants exposed to the supernatural visualization appraised their squad as having performed more skillfully than the opposing squad (Table 1). Our exploratory analysis of potential effects of the manipulation on perceptions of personal performance relative to their fellow squad members revealed no significant difference between players in the supernatural condition (*M* = 6.23; *SD* = 1.54) and the control condition (*M* = 5.38; *SD* = 1.86, *p* = .099, η^*2*^_*p*_ = .06 [95% CI = −.17, 1.87]).

### Heightened Appraisal of Coalitional Performance Mediates Heightened Appraisal of Personal Performance Following Supernatural Visualization

Post-battle appraisals of personal and coalitional performance were strongly correlated, *r*_44_ = .48, *p* = .001. Therefore, to assess the extent to which the observed increase in post-battle assessments of personal performance in the supernatural support condition was explained by the covarying increase in appraisals of coalitional performance, we next conducted a mediation test. We utilized the bias-corrected bootstrapping procedure (5000 samples) found in the INDIRECT macro for SPSS (Preacher and Hayes 2008). We entered the visualization condition as the independent variable, appraisal of coalitional performance as the mediating variable, and appraisal of personal performance as the dependent variable. Indeed, the direct effect of the supernatural visualization on appraisals of personal performance (*b* = −1.61, *SE* = .51, β = −.43, *p* = .003) was no longer significant in the model (*b* = −.96, *SE* = .56, β = −.26, *p* = .092), whereas the indirect effect of appraisals of coalitional performance on appraisals of personal performance remained significant (*b* = .32, *SE* = .14, β = .35, *p* = .026), although the confidence intervals did slightly overlap with zero (95% CI = −1.44, .04). In contrast, in a model including appraisals of personal performance as a prospective mediator and coalitional performance as the outcome variable, the direct effect of the supernatural visualization on appraisals of coalitional performance remained significant (*b* = −1.44, *SE* = .55, β = −.36, *p* = .012). In sum, the post-battle perceptions of enhanced personal performance in the supernatural visualization condition were accounted for by perceptions of enhanced coalitional performance, whereas the effect of the supernatural visualization on appraisals of coalitional performance were independent of effects on perceived personal performance.

### Null Effect of Supernatural Visualization on Self-Reported Risk-Taking during Battle

A binary logistic regression revealed no significant difference between players in the supernatural condition (*M* = .32; *SD* = .48) and the control condition (*M* = .29; *SD* = .46) in self-reported risk-taking during the battle, *p* = .845.

### Checks for Potential Confounding Differences in Battle Outcomes

As noted above, no squad successfully captured the opposing squad’s flag during data collection; hence, responses concerning subjective perceptions of winning were not confounded by objective experiences of victory or defeat. Also as intended, there was no difference between conditions in the number of opposing players that participants reported having shot, *p* = .750, further ensuring that the effects of the supernatural visualization on post-battle confidence were driven by subjective perceptions rather than by objective differences in performance.

In an effect most likely owing to chance, a binary logistic regression revealed that significantly fewer players in the supernatural support condition reported being shot during the battle (0 = Not shot; 1 = Shot; *M* = .27; *SD* = .46) relative to the players in the control condition (*M* = .63; *SD* = .50), *p* = .019. (Note that the null effect of condition on number of opponents reported shot can readily be reconciled with an effect of condition on the number of players reporting having been shot themselves because players may have been shot by multiple opponents and, given the chaotic nature of paintball battles, players cannot always be certain whether they have hit their targets.) Follow-up analyses confirmed that controlling for whether a player reported being shot during the battle does not alter any of the statistically significant effects of condition on post-battle confidence, nor does it substantively reduce the observed effect sizes (η^2^_*p* =_ 10–.21; compare with models not including this covariate, η^2^_*p*_ = 16–.27, see Table 1).

Finally, we ran a series of exploratory partial correlations, controlling for visualization condition, to assess potential associations between reports of being shot, the number of enemies reported shot, self-reported risk-taking during the battle, and both pre-battle performance predictions and post-battle performance appraisals (see ESM, Table S2).

## Discussion

Our results indicate that perceptions of supernatural support may promote willingness to engage in intergroup conflict due to enhanced battle confidence. We primed participants to visualize either supernatural support or a control topic immediately prior to engaging in coalitional combat as simulated within a play context. Consistent with predictions, participants in the supernatural support condition reported greater confidence in their team’s prospects for victory prior to battle, retrospectively assessed their team’s performance during the battle as superior to that of the opposing team, and regarded their team as more likely to achieve victory in a future rematch. Overall, these results align with anecdotal real-world observations of a relationship between religiosity and willingness to engage in violent conflict. The present findings regarding coalitional battle confidence likewise extend the emerging body of work relating religious cognition with aggression (e.g., Atran and Ginges 2012; Kruglanski et al. 2009; McKay et al. 2011) and with optimistic perceptions of formidability relative to one’s adversaries (Holbrook et al. 2016a; Sosis et al. 2012).

Participants in the supernatural support condition also retrospectively assessed their personal performance in the battle as superior to that of their opponents. On the one hand, this result agrees with prior findings that cues of supernatural support can heighten confidence in one’s personal chances of victory (Holbrook et al. 2016a). On the other hand, the effect of the supernatural manipulation on personal confidence was accounted for by concomitant elevation in coalitional confidence, suggesting that perceptions of having personally performed better in the battle were driven by participants’ perceptions that they had done so only insofar as they were embedded in a supernaturally supported team, an interpretation that is reinforced by the finding that participants did not assess their personal performance as significantly superior to that of their teammates. The specificity of the effects of the supernatural manipulation with respect to boosting confidence in personal performance relative to enemies, but not to fellow teammates, is consistent with a potential elevation of coalitional over individual identity among the players, as a sense of coalitional entitativity is related to perceived ability to triumph in battle (Fessler and Holbrook 2016), and since individuals whose affiliations might otherwise diverge tend to unite in opposition to rival coalitions (Kurzban and Neuberg 2005). Although we did not measure entitativity directly, in this regard it is important to note that the quasi-ritual nature of the visualization exercise was constant across the two conditions. Accordingly, any effects of the supernatural manipulation on coalitional confidence via changes in entitativity are presumably due exclusively to the content of the visualization, and not to such factors as the experience of kneeling together (see, for example, Fischer et al. 2013), which could plausibly enhance entitativity independent of supernatural content.

Our observations with regard to the effect of the manipulation on personal versus coalitional confidence should not be taken as conclusive evidence that the effects of perceived supernatural support are limited to or entirely driven by coalitional psychology. The experiences of squad assignment and of combat in our modified version of Capture the Flag were inherently coalitional and may have shaped perceptions of supernatural support and of victory accordingly. Had we instead provided individuals an opportunity to fight individually, we may well have detected an effect of the supernatural support manipulation on personal confidence independent of coalitional reckoning. Future research should explore this possibility, and the more general question of the relevance of particular modes of combat to the effects of perceived supernatural support, by varying the nature of the simulated conflict.

Against expectations, and despite a significant increase in participants’ pre-battle anticipation of overall team victory, we observed no effects of the supernatural support manipulation on anticipated pre-battle coalitional or personal performance. These null results diverge notably from the large, consistent effects that we observed with regard to the ratings of coalitional and personal performance and anticipated future victory collected immediately following the battle (see Table 1). Speculatively, the finding that participants in the supernatural condition were more confident of victory suggests that, before the battle, the prospective advantage may have been conceptualized as independent of enhanced personal or coalitional battle performance (i.e., some sort of divine intervention). Alternatively, potential pre-battle effects of the supernatural support prime may have been swamped by the near-ceiling levels of pre-battle confidence observed in both conditions (see Table 1). Interestingly, when asked to retrospectively appraise their coalitional and personal performance after the battle, ratings provided by participants in the control condition hover just above the middle of the scale (see Table 1), suggesting that these participants’ pre-battle confidence may have been tempered by the actual experience of battle, including being pinned down, having difficulty hitting targets, and ultimately failing to capture the enemy’s flag. In contrast, participants in the supernatural support condition appear not to have integrated such deflating experiences into their post-battle appraisals, possibly suggesting that the expectation of supernatural support engendered a positivity bias coloring either their perceptions during battle, their recollections after battle, or both. Alternately, a more radical possibility that the present self-report data cannot rule out is that, rather than being under the sway of a positivity bias, the participants in the supernatural support condition may have actually performed better during battle. Although no team objectively won in the sense of capturing the opposing flag, it may be the case that perceptions of supernatural support enhanced combat performance in some respects, as hinted at by the fact that participants in the supernatural support condition reported suffering fewer simulated fatalities despite reporting an equivalent level of risk-taking to that described by participants in the control condition. Although worth examining in the future by objectively assessing battle performance behavior, this conjecture should be approached with great caution as (*a*) the between-group disparity in reports of being shot may be due to chance given our limited sample size, (*b*) there was no effect of condition on the self-reported number of enemies shot, and (*c*) the effects of the supernatural support manipulation on post-battle confidence withstood controlling for reports of being shot during battle.

Just as the present results call for follow-up studies assessing the effects of perceived supernatural support on actual combat performance, they also invite further behavioral research on the relationship between perceived supernatural support and nonconflictual forms of physical risk-taking (e.g., skydiving). Subtle cues of supernatural support have been found to diminish perceptions of the self as likely to suffer injuries and to increase willingness to take hypothetical nonviolent physical risks (Kupor et al. 2015) and financial risks (Chan et al. 2014). When these findings are synthesized with the present results, perceived supernatural support appears to prime various forms of risk-tolerance, from nonviolent expressions of bravery despite physical hazard to violent behavior in conflictual contexts.

The visualization exercise and subsequent paintball contest utilized in this study engaged participants in direct proxies for both group prayer and coalitional combat. Although this face-valid approach to the relevant phenomenon offers clear translational benefits, such a straightforward strategy may also be susceptible to demand characteristics. However, the present findings do not appear explicable as artifacts of demand characteristics. Had participants reported greater battle confidence following the supernatural prime on the basis of demand characteristics, they would presumably have reported comparably heightened confidence both before and after the battle, whereas the great majority of the effects of our manipulation appear only in the post-battle ratings. Our reliance on a community sample of weekend paintball players also mitigates the risk of demand effects as there is no reason to suspect that our sample was versed in research techniques.

The present sample’s characteristics raise considerations with regard to the generalizability of the results. To begin, the participants in our field study self-selected to engage in competitive team paintball. On the one hand, this aspect of the sample arguably implies the presence of individual differences which might covary with tendencies to engage in actual conflict, thus increasing the translational validity of these results. On the other hand, the presumable presence of self-selecting factors relevant to seeking out aggressive sports such as paintball raises the possibility that the observed effects may not obtain in samples lacking such factors. In addition, our study utilized an entirely male sample. Although there are no evident grounds to suspect that women would respond differently, men may display a more pronounced response to cues of supernatural aid in battle, as men are vastly overrepresented in combat-related sports (Apostolou 2015; Deaner and Smith 2013) and are more sensitive to factors relevant to intergroup conflict, in keeping with their greater ancestral role in coalitional aggression (McDonald et al. 2012). Future work should assess the potential role of sex in moderating the influence of supernatural support cues on combat confidence. Finally, the present sample derives from a WEIRD society (Henrich et al. 2010) in which supernatural beliefs regarding benevolent, supportive agents are common—precisely the sort of sample for which cues of supernatural aid should heighten battle confidence. The present findings may not generalize to societies lacking such beliefs or that predominantly associate supernatural agents with hazard or malevolence (Holbrook and Sousa 2013). Cross-cultural work on perceived supernatural support and battle confidence must be sensitive to local conceptions of the supernatural, and modify the priming stimuli (which we tailored to our WEIRD sample), battle simulations, and dependent measures accordingly.

The present data indicate that perceived supernatural support can facilitate tendencies to engage in conflict by enhancing battle confidence. Future work should explore the theoretically complementary role of perceived supernatural monitoring of moralized behavior with regard to in-group cooperation, self-sacrifice, and norm-adherence, all of which appear directly relevant to willingness to fight and defeat adversarial groups (Atran and Ginges 2012; Richerson et al. 2016; Roes and Raymond 2003).

We experimentally manipulated confidence in a form of competitive team play, yielding effects convergent to those hypothesized to pertain to actual combat (e.g., Holbrook et al. 2016b). Thus, in line with the methodological premise motivating the study, paintball does appear to evoke the psychology of coalitional violence, and thereby to invoke responsiveness to factors hypothesized to influence actual combat cognition, such as perceived supernatural support. Although this may seem unremarkable given that paintball is contrived to simulate warfare, on reflection it is noteworthy that groups of adult men playing together with paint markers in a field, all of whom know that they face no real hazard, appear to subjectively experience their play in a manner akin to that of warring coalitions suffering and inflicting injury and death. This fact of human psychology suggests that competitive group play-fighting may indeed serve an adaptive function with regard to actual coalitional combat.

Play appears closely linked with the development of flexible, complex skills, given that play behavior is most common in species with relatively large and complex neocortices, typically peaks during periods of maximal cortical development, and is most frequently observed in the young (Chick 2001; Fagen 1974), all of which point to a preparatory function of group play-fighting (Boulton and Smith 1992). However, functions related less to refining combat skills and more to facilitating adaptive social bonding or coordination also appear plausible and should be tested in future work. If play-fighting were not adaptive in some regard, particularly in light of the risk of injury intrinsic to rough physical play, individuals that expended precious time and energy in play-fighting would be at a selective disadvantage relative to individuals directing their efforts toward fitness-enhancing endeavors (Symons 1978).

## Conclusion

The results of our field study of coalitional paintball combat are consistent with the hypothesis that perceptions of supernatural support can potentiate both prospective confidence in winning battles and retrospective confidence in the battle performance of oneself and one’s team. These findings hold unambiguous relevance to such real-world phenomena as religious extremism. The success of these methods arguably also illustrates the social function of competitive team sports as playful proxies of violent group conflict.

## Electronic supplementary material

ESM 1 (PDF 271 kb)

## Footnotes

1 Two participants completed every measure in both surveys with the sole exception of the item in the post-battle packet probing perceptions of victory. Rather than drop these two individuals from the sample, the modal response was inserted. Follow-up tests confirm that dropping these two participants does not change the overall pattern of results.

2 We also included a novel, exploratory behavioral measure of pre-battle confidence in the form of a raffle wherein players could wager their tickets on their squad winning the game (see ESM for details). However, discussions with players during the study (i.e., many asking clarification questions about the raffle after the pre-battle survey had been turned in) as well as afterwards indicated that the instructions were overly complicated and poorly understood by many participants. Accordingly, this raffle measure is not discussed further.

3 This study was conducted under field conditions far more distracting than those typical of conventional laboratory research. While deleterious in obvious respects (e.g., the lack of completed demographics packets) and likely to have introduced some degree of methodological as well as literal background noise, these somewhat hectic conditions arguably added to the verisimilitude of the study conditions as a model of actual group conflict.
