# Supplementary material for: May God Guide Our Guns: Visualizing Supernatural Aid Heightens Team Confidence in a Paintball Battle Simulation
Source: Hum Nat. 2018 Jun 18;29(3):311–27. doi: 10.1007/s12110-018-9320-8 (PMC6132840; doi:10.1007/s12110-018-9320-8)
Supplement: Supplementary file 1 — (PDF 271 kb) [file 12110_2018_9320_MOESM1_ESM.pdf]

Electronic Supplementary Materials

May God Guide Our Guns:
Visualizing Supernatural Aid Heightens Team Confidence in a Paintball Battle Simulation

Jeremy Pollack,* Colin Holbrook,* Daniel M.T. Fessler, Adam Sparks, & James G. Zerbe

* equal contribution

Human Nature 29(3), 2018, https://doi.org/10.1007/s12110-018-9320-8

Contents

Figure S1. Participants receiving study instruction.

Table S1. Descriptive statistics for measures of state affect.
Table S2. Partial correlations (controlling for visualization condition) between pre- and post-battle confidence measures and reports of participant being shot, the number of enemies reported shot by participant, and risk-taking during the battle.

Visualization Scripts

Pre-Battle Survey

Post-Battle Survey

Exploratory Raffle Measure

The complete dataset, syntax files, and visualization stimuli are archived at
https://osf.io/e9d68/?view_only=85cba56aba3e4a7cbe9d651d5f654d9e

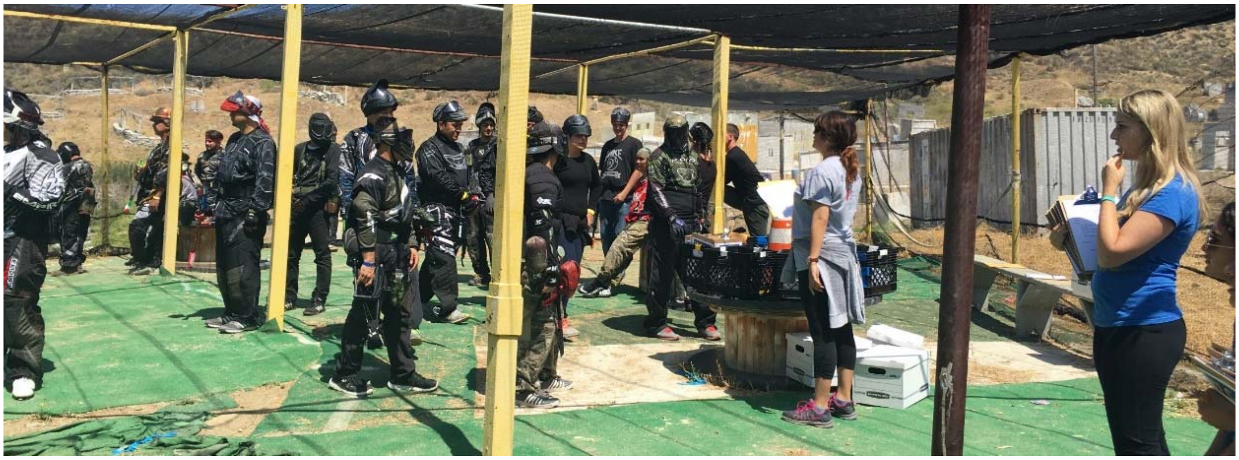

Figure ESM1. Participants receiving study instruction.

Table S1. Descriptive statistics for measures of state affect ($N = 46$)

	Control *Mean (SD)*	*Supernatural Mean (SD)*
Pre-battle 'nervousness'	3.17 (2.32)	2.55 (2.06)
Post-battle 'nervousness'	3.17 (2.30)	3.00 (2.39)
Pre-battle 'excitement'	6.71 (1.52)	6.82 (1.30)
Post-battle 'excitement'	6.29 (1.57)	6.27 (1.49)

Table S2. Partial correlations (controlling for visualization condition) between pre- and post-battle confidence measures and reports of participant being shot, the number of enemies reported shot by participant, and risk-taking during the battle

	Participant Shot	No. of Enemies Shot	Risk-taking during Battle
Pre-Battle			
Anticipated victory	−.18	.00	.31*
Personal > enemies	−.15	.29†	.27†
Coalitional performance	−.08	.14	.26†
Personal > squadmates	−.20	.15	.13
Post-Battle			
Perceived victory	−.20	−.04	−.10
Rematch confidence	−.06	.03	.34*
Personal > enemies	−.39**	.40**	−.02
Coalitional performance	−.14	.07	.05
Personal > squadmates	−.14	.41**	−.06

† $p < .10$, * $p < .05$, ** $p < .01$. Participant Shot (0 = No; 1 = Yes); Risk-taking during Battle (0 = Take cover and target enemies; 1 = Cross open ground to help capture enemy flag).

Visualization Scripts

(Audio stimuli are archived at https://osf.io/e9d68/?view_only=85cba56aba3e4a7cbe9d651d5f654d9e**)**

God Visualization Text

Please close your eyes. Take three deep breaths. One… Two… Three… Good. Now I want you to imagine yourself on the field, your gun in your hand, ready to fight. Now imagine that there's a powerful force with you. You might call that force God or spirit or the universe or maybe even just the power of intention. Whatever that higher power is for you, imagine that it is with you now, that it's by your side, that it's within you, in every part of you. Imagine that power guiding your hands and your eyes, guiding your trigger steady, protecting you as you move through the game. Know that this powerful guide is with you, and with your team, helping your team, wanting your team to win. What does this powerful energy feel like? Feel it deeply within and all around you. It's protecting you, it's guiding you, it is yours. Take a few more moments now to feel this power, to feel this force. And on the count of three, I want you to slowly open your eyes. One... Two... Three... Okay, slowly open your eyes, and rise to your feet.

Control Visualization Text

Please close your eyes. Take three deep breaths. One… Two… Three… Good. Now I want you to imagine yourself on the field, your gun in your hand, ready to fight. Now imagine that you are standing next to a tree. The tree might be an oak, or a pine, or a eucalyptus, or whatever sort of tree you prefer. Whichever tree you prefer, imagine standing next to it. The tree is by your side. Imagine what the bark looks like. Imagine what the leaves look like. Imagine touching the tree. What does it feel like? Now take a few more moments to imagine this tree. And on the count of three, I want you to slowly open your eyes. One... Two... Three... Okay, slowly open your eyes, and rise to your feet.

Pre-Battle Survey

- How confident are you that your squad will WIN (i.e., capture the flag)?

  Not at All ☐ ☐ ☐ ☐ ☐ ☐ ☐ ☐ *Extremely*

- How confident are you that your squad will NOT LOSE (i.e., win or draw)?

  Not at All ☐ ☐ ☐ ☐ ☐ ☐ ☐ ☐ *Extremely*

- How well do you feel YOU will perform in game compared with members of the opposing squad?

  Not at All ☐ ☐ ☐ ☐ ☐ ☐ ☐ ☐ *Extremely*

- How well do you feel YOU will perform in game compared with the other members of your squad?

  Not at All ☐ ☐ ☐ ☐ ☐ ☐ ☐ ☐ *Extremely*

- How well do you feel your SQUAD will perform compared with the opposing squad?

  Not at All ☐ ☐ ☐ ☐ ☐ ☐ ☐ ☐ *Extremely*

- How excited or “into it” do you feel?

  Not at All ☐ ☐ ☐ ☐ ☐ ☐ ☐ ☐ *Extremely*

- How nervous do you feel?

  Not at All ☐ ☐ ☐ ☐ ☐ ☐ ☐ ☐ *Extremely*

Post-Battle Survey

- In the battle, did your squad Win, Lose, or Draw?

WIN LOSE DRAW

- How well do you feel that YOU performed in the game compared with members of the opposing squad?

Not at All ☐ ☐ ☐ ☐ ☐ ☐ ☐ ☐ *Extremely*

- How well do you feel that YOU performed in the game compared with the other members of your squad?

Not at All ☐ ☐ ☐ ☐ ☐ ☐ ☐ ☐ *Extremely*

- How well do you feel that your SQUAD performed compared with the opposing squad?

Not at All ☐ ☐ ☐ ☐ ☐ ☐ ☐ ☐ *Extremely*

- How excited or "into it" did you feel during the battle?

Not at All ☐ ☐ ☐ ☐ ☐ ☐ ☐ ☐ *Extremely*

- How nervous did you feel during the battle?

Not at All ☐ ☐ ☐ ☐ ☐ ☐ ☐ ☐ *Extremely*

- If there were a rematch, how confident are you that your squad would win?

Not at All ☐ ☐ ☐ ☐ ☐ ☐ ☐ ☐ *Extremely*

- During the battle, which strategy did you use the most? (Select one):
    - *Take cover and target opponents*
    - *Cross open ground to capture the enemy flag*

- During the battle, how many members of the opposing force do you think you shot? (Select one):
    - *None*
    - *1*
    - *2*
    - *3*
    - *4*
    - *5*
    - *6*
    - *7*
    - *More than 7*

- Did you get shot during the battle?

    - o *No*
    - o *Yes*

Exploratory Raffle Measure

You have an opportunity to gamble on **BEATING** the opponent team.

If your team **WINS**, you will **GAIN** the number of raffle tickets you choose to gamble.

If your team **LOSES**, you will **LOSE** the raffle tickets you choose to gamble.

This is a **REAL DECISION**. After the game, you will either **GAIN** or **LOSE** the number of tickets you wager.

Please **circle one option** below to indicate how many of your raffle tickets you would like to gamble:

0 tickets

1 ticket

2 tickets

3 tickets

4 tickets

5 tickets

Please select your preference for the **SIZE** of the opponent team, if it were up to you.

The **SMALLER** the opponent team you choose, the **LESS** you would gain if your team wins.
The **LARGER** the opponent team you choose, the **MORE** you would gain if your team wins.

Please **circle the option** that you would prefer:

**Opponent Team Size:**	**Raffle Outcome if you Win:**
**Option A:** Same as Your Team	0 Extra Tickets
**Option B:** One More than Your Team	2 Extra Tickets
**Option C:** Two More than Your Team	4 Extra Tickets
**Option D:** Three More than Your Team	6 Extra Tickets
**Option E:** Four More than Your Team	8 Extra Tickets
